# Rapid research response to the COVID-19 pandemic: perspectives from a National Institute for Health Biomedical Research Centre

**DOI:** 10.1186/s12961-022-00827-0

**Published:** 2022-02-19

**Authors:** Lorna R. Henderson, Helen McShane, Vasiliki Kiparoglou

**Affiliations:** 1 National Institute for Health Research Oxford Biomedical Research Centre, John Radcliffe Hospital, Oxford, United Kingdom; 2 Radcliffe Department of Medicine, University of Oxford, Oxford, Oxford United Kingdom; 3 Nuffield Department of Medicine, University of Oxford, Oxford, Oxford United Kingdom; 4 Nuffield Department of Primary Health Care Sciences, University of Oxford, Oxford, United Kingdom

**Keywords:** COVID-19, Biomedical Research Centres, Translational research, Organizational resilience, Rapid research response

## Abstract

With over 5 million COVID-19 deaths at the time of writing, the response of research leaders was and is critical to developing treatments to control the global pandemic. As clinical research leaders urgently repurposed existing research programmes and resources towards the COVID-19 pandemic, there is an opportunity to reflect on practices observed in Biomedical Research Centre (BRC) settings. BRCs are partnerships between leading National Health Service organizations and universities in England conducting translational research for patient benefit funded by the National Institute for Health Research (NIHR). Oxford BRC-supported researchers have led the rapid set-up of numerous COVID-19 research studies at record speed with global impact. However, the specific contribution of BRCs to the COVID-19 pandemic in the literature is sparse. Firstly, we reflect on the strategic work of clinical research leaders, creating resilient NIHR research infrastructure to facilitate rapid COVID-19 research. Secondly, we discuss how COVID-19 rapid research exemplars supported by Oxford BRC illustrate “capacity”, “readiness” and “capability” at an organizational and individual level to respond to the global pandemic. Rapid response research in turbulent environments requires strategic organizational leadership to create resilient infrastructure and resources. The rapid research exemplars from the Oxford BRC illustrate capability and capacity at an organizational and individual level in a dynamic environment to respond during the COVID-19 public health challenge. This response was underpinned by swift adaptation and repurposing of existing research resources and expertise by the Oxford BRC to deliver rapid research to address different aspects of COVID-19.

## Background

With over 5 million COVID-19 deaths at the time of writing, the response of research leaders was and is critical to developing treatments to control the global pandemic [1]. As clinical research leaders have urgently repurposed existing research programmes and resources towards the COVID-19 pandemic, there is an opportunity to reflect on practices observed in Biomedical Research Centre (BRC) settings. BRCs are partnerships between leading National Health Service (NHS) organizations and universities in England conducting translational research for patient benefit funded by the National Institute for Health Research (NIHR) [2, 3]. Oxford BRC-supported researchers have led the rapid set-up of numerous COVID-19 research studies at record speed with global impact [4]. However, the specific contribution of BRCs to the COVID-19 pandemic in the literature is sparse [5–9]. Firstly, we reflect on the strategic work of clinical research leaders, creating resilient NIHR research infrastructure to facilitate rapid COVID-19 research. Secondly, we discuss how COVID-19 rapid research exemplars supported by Oxford BRC illustrate “capacity”, “readiness” and “capability” at an organizational and individual level to respond to the global pandemic.

### BRCs

BRCs were established in 2007, bringing world-leading NHS clinicians and university academics together to conduct “translational research”, developing groundbreaking treatments for patient benefit as well as the NHS [2]. There are now 20 BRCs, and this source of funding enables supported researchers to develop innovative research but also to attract external investment contributing to the health and wealth of the nation [2].

During the COVID-19 pandemic, BRC researchers have been at the forefront of the rapid research response, exemplified by the development and testing of vaccines and early-phase drug trial at record speed [4]. Reflecting back, Professor Dame Sally Davies’ strategic leadership as the “architect” of the NIHR in 2006 created strong research infrastructure and significant funding (> £1 billion annual budget), securing the United Kingdom’s reputation as a world leader in clinical research [10–12]. The foresight in creating this translational research funding and infrastructure enabled an agile response in times of crisis, including the development of an effective vaccine against SARS-CoV-2 [2–4, 7, 10–12]. This new source of funding catalysed significant expansion in the capacity and capability of translational research and the establishment of BRCs [2, 10]. The importance of research funding and infrastructure to respond to the COVID-19 pandemic is recognized, including the role of the United Kingdom Clinical Research Networks in rapid study set-up and recruitment [8, 9, 13]. Comparisons between the Canadian clinical research system and the United Kingdom NIHR underline the importance of not only adequate research funding but, crucially, integration with the NHS to facilitate agile response to crises [13].

### Oxford BRC

**Fig. 1:**
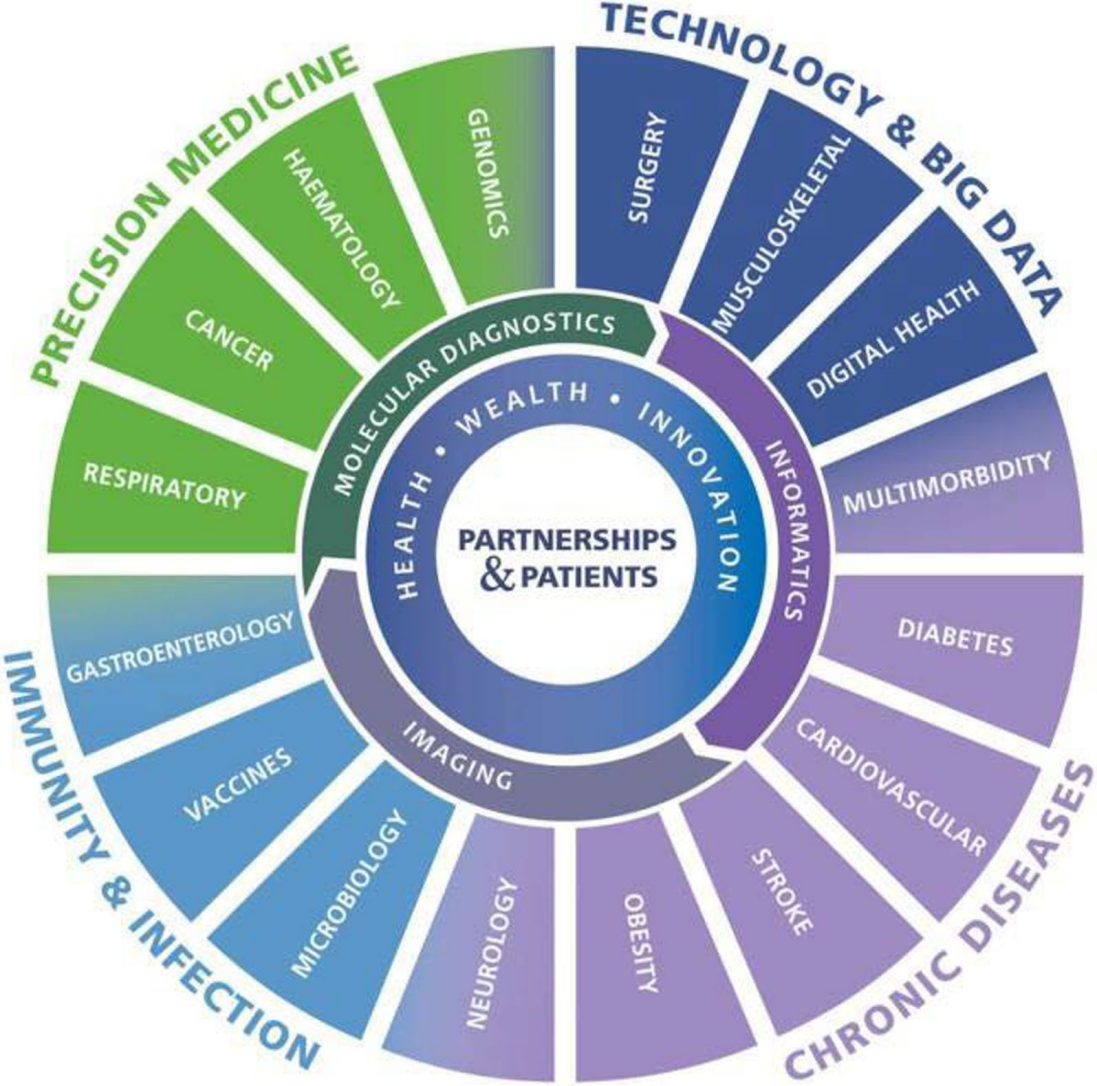
Oxford BRC research themes and clusters

Oxford BRC is a partnership between Oxford University Hospitals NHS Foundation Trust, which has an international reputation for clinical research, and the University of Oxford, currently ranked as the world’s best institution for medical teaching and research [14, 15]. The Oxford BRC supports world-leading NHS clinicians and academics across 20 research themes (Fig. 1) [4]. It was well placed to deliver rapid COVID-19 research, as it is characterized by partnership working: many of the researchers are practising clinicians providing direct care to patients and the public, alongside their academic work. Furthermore, Oxford BRC research is fully integrated with Oxford’s hospitals, ensuring medical innovations can be moved quickly “from bench to bedside”, out of laboratories into clinical trials and onto the NHS care setting [4].

### Delivery rapid response COVID-19 research

Here we illustrate how the Oxford BRC supported a rapid response into COVID-19 research, demonstrating “capacity”, “readiness” and “capability” at an organizational and research leadership level in a dynamic environment [4, 16]. At the beginning of the pandemic, Oxford BRC research funding was quickly reallocated to “pump-prime” emerging high-impact COVID-19-related research, including the Oxford/AstraZeneca (AZ) COVID-19 vaccine and the RECOVERY trial [7, 17, 18]. Over 1 year later, Oxford BRC has supported over 100 COVID-19 projects, 34 of which were Urgent Public Health (UPH) research studies nationally prioritized by NIHR [19]. COVID-19 rapid research required agile, collaborative leadership behaviour but also innovative high-performing teams who can quickly adapt and implement complex research at speed [4, 7]. This is illustrated in our Vaccines Theme with the development of the Oxford/AZ SARS-CoV-2 vaccine.

### Exemplars of Oxford BRC COVID-19 rapid research

#### The Oxford/AZ COVID-19 vaccine

Oxford BRC provided crucial early funding to expedite the development of the COVID-19 vaccine work led by the Vaccines Theme researchers and then contributed to a clinical trial to evaluate the safety of the vaccine [4, 7]. The Vaccines Theme researchers used their expertise and existing BRC-funded infrastructure to rapidly develop a vaccine for SARS-CoV-2. When the SARS-CoV-2 virus emerged in China at the end of 2019, Vaccines Theme researchers were already working on human coronavirus vaccines and were therefore in a unique position to respond rapidly to the pandemic. Professor Andrew Pollard, co-Theme Lead for the Vaccines Theme, led the phase III testing of this vaccine candidate across 19 trial sites in the United Kingdom, South Africa and Brazil. Work to develop this vaccine first began in January 2020, and the vaccine was approved in January 2021 at unprecedented speed [17].

#### The RECOVERY trial

Funding from the Oxford BRC and others contributed to the Randomised Evaluation of COVID-19 Therapy (RECOVERY) trial, providing evidence about potential treatments for those hospitalized with COVID-19 or suspected COVID-19 [18, 20]. The trial was led by Professor Peter Horby and Professor Martin Landray, Theme Lead for the Clinical Informatics and Big Data cross-cutting Theme [4, 18]. This UPH trial was rolled out at record speed, with the first patient being enrolled just 9 days after the protocol was first drafted [20]. Over 10,000 patients were recruited within 8 weeks, and the results from the first arm were announced 12 weeks later. A total of 176 acute hospital trusts (including all 20 of the BRCs) across the four United Kingdom nations are participating in this trial. It was and is the world’s largest randomized controlled trial of treatments for patients hospitalized with COVID-19 and has already identified two effective treatments [18].

## Conclusion

The rapid research exemplars from the Oxford BRC illustrate capability and capacity at an organizational and individual level in a dynamic environment to respond during the COVID-19 public health challenge [4, 7, 16]. This response was underpinned by swift adaptation and repurposing of existing research resources and expertise by the Oxford BRC to deliver rapid research to address different aspects of COVID-19 [4, 7]. Rapid response research in turbulent environments requires strategic organizational leadership to create resilient infrastructure and resources.

## Data Availability

Data sharing is not applicable to this article as no datasets were generated or analysed.
